# Experimental Vaccines against Chagas Disease: A Journey through History

**DOI:** 10.1155/2015/489758

**Published:** 2015-05-18

**Authors:** Olivia Rodríguez-Morales, Víctor Monteón-Padilla, Silvia C. Carrillo-Sánchez, Martha Rios-Castro, Mariana Martínez-Cruz, Alejandro Carabarin-Lima, Minerva Arce-Fonseca

**Affiliations:** ^1^Department of Molecular Biology, Instituto Nacional de Cardiología Ignacio Chávez, Juan Badiano No. 1, Colonia Sección XVI, 14080 Delegación Tlalpan, DF, Mexico City, Mexico; ^2^Centro de Investigaciones Biomédicas, Universidad Autónoma de Campeche, Avenida Agustín Melgar No. 3, 24030 Campeche, CAM, Mexico; ^3^Centro de Investigaciones en Ciencias Microbiológicas, Benemérita Universidad Autónoma de Puebla, 14 Sur y Avenida San Claudio, Ciudad Universitaria, 72570 Puebla, PUE, Mexico

## Abstract

Chagas disease, or American trypanosomiasis, which is caused by the protozoan parasite *Trypanosoma cruzi*, is primarily a vector disease endemic in 21 Latin American countries, including Mexico. Although many vector control programs have been implemented, *T. cruzi* has not been eradicated. The development of an anti-*T. cruzi* vaccine for prophylactic and therapeutic purposes may significantly contribute to the transmission control of Chagas disease. Immune protection against experimental infection with *T. cruzi* has been studied since the second decade of the last century, and many types of immunogens have been used subsequently, such as killed or attenuated parasites and new DNA vaccines. This primary prevention strategy appears feasible, effective, safe, and inexpensive, although problems remain. The objective of this review is to summarize the research efforts about the development of vaccines against Chagas disease worldwide. A thorough literature review was conducted by searching PubMed with the terms “Chagas disease” and “American trypanosomiasis” together with “vaccines” or “immunization”. In addition, reports and journals not cited in PubMed were identified. Publications in English, Spanish, and Portuguese were reviewed.

## 1. Introduction

Chagas disease, or American trypanosomiasis, is caused by the protozoan parasite* Trypanosoma cruzi*. Chagas disease is primarily a vector disease endemic in 21 Latin American countries, where it has a strong economic impact because it primarily affects economically active people. Approximately 10 million people are infected, and >25 million people are at risk of infection in endemic countries [1].

After years or decades that infection is acquired, from 10% to 30% of infected people develop symptoms of chronic phase. The heart is the most commonly affected organ; symptoms include arrhythmias, cardiomyopathy, and thromboembolism. Death usually occurs from heart failure [2].

Although vector control and blood bank serological screening have greatly reduced parasite transmission, the costs to maintain such control programs as well as the differences between vector species, the existence of animal reservoirs, the parasite persistence in chronically infected people, and lack of adequate chemotherapy for the treatment of infection have made the complete eradication of* T. cruzi* impossible. An additional measure which could significantly contribute to the control of this disease is the development of an anti-*T. cruzi* vaccine [3–5]. The immunological protection against experimental infection with* T. cruzi* has been studied since the second decade of the last century and many types of immunogens such as killed or attenuated parasites and the newest DNA vaccines have been tested.

## 2. History

Since 1912, when Blanchard demonstrated that animals surviving acute infection with* T. cruzi* became resistant to reinfection, active immunization against this protozoan began to be researched. Blanchard's experiments were confirmed by Brumpt, Mayer, and Rocha-Lima who used trypanosome blood forms for their studies [6].

In 1952, for the first time, Pizzi and Prager used cultured parasite attenuated forms to protect animals against infection with a* T. cruzi* virulent strain. Two years later, Rubio showed that infection with these cultured forms was exacerbated by corticosteroids. Various and partial results were obtained when live attenuated forms were tested in other immunization attempts of laboratory animals [7]. Immunization with dead trypanosomes began in 1912 with Laveran. Since then, several chemical and physical methods to kill parasites were evaluated, always with unsatisfactory results, except for those of Goble and colleagues who used a pressure chamber to dislodge the parasite and generate a vaccine [6].

González Cappa and colleagues observed that 88% and 100% of the animals, depending on the dosage and number of immunizations, were protected using antigens prepared in a pressure chamber [8].

In 1968, Menezes determined that the Y strain cultured for 15 years became avirulent, most likely because of mutations [9]. It was experimentally demonstrated that this strain protected animals against infection with different* T. cruzi* strains, suggesting that virulence factors are not essential as immunogens.

## 3. Live, Killed, or Attenuated Parasite Immunization

Live trypanosome immunization as well as killed or attenuated parasites for the preparation of immunogens that use physical or chemical methods has been performed. A brief overview of this type of immunization is presented below.

Epimastigotes of* Trypanosoma rangeli* fixed with glutaraldehyde and emulsified with saponin as an adjuvant were injected by laboratory of Introini. Mice were infected with 100 trypomastigotes of the Tulahuén* T. cruzi* strain [10]. The results showed that 2 or 3 doses are required to induce a significant reduction in parasitemia and increase survival.

Apart from* T. rangeli*, other trypanosomatids have been used as vaccines against* T. cruzi*. Breganó and colleagues immunized BALB/c mice either intraperitoneally or orally with* Phytomonas serpens*, a tomato parasite that shares antigens with* T. cruzi*, and after lethal challenge with trypomastigotes, the mice showed a significant decrease in parasitemia and increased survival [11].

Inducible nitric oxide synthase (iNOS)* knockout* mice and C57BL/6 mice treated with an iNOS inhibitor were immunized orally with* Phytomonas serpens* and challenged with* T. cruzi*. A reduction in parasitemia and increased survival of C57BL/6 mice compared to the* knockout* animals were observed [12], suggesting that nitric oxide is a mechanism of parasite control.

Basso and colleagues vaccinated BALB/c mice with live or fixed epimastigotes of two* T. rangeli* strains and lower levels of parasitemia and an increased survival rate were observed after* T. cruzi* Tulahuén strain infection. Histology revealed a moderate lymphocytic infiltrate [13]. This study demonstrated that the antigens involved in the protection induced by* T. rangeli* are expressed in different strains of this parasite, suggesting that it may be useful in the preparation of vaccines. Years later they immunized guinea pigs with epimastigotes of* T. rangeli* emulsified with saponin and subsequently challenged them with Tulahuén strain of* T. cruzi* trypomastigotes. These guinea pigs showed significantly lower parasitemia and a discrete lymphocytic infiltrate in the cardiac and skeletal muscles. In the chronic phase, the histology was normal. The control group exhibited nests of amastigotes and histopathological changes compatible with chagasic myocarditis, endocarditis, and pericarditis [14]. The immunoprotection by* T. rangeli* was shown and this identified new possible preventive tools that may reduce the risk of infection with* T. cruzi*.

In addition to attenuated parasites, animal models have also been immunized with mutant strains. The research group of Basombrio generated monoallelic mutant parasites for the* dhfr-ts* gene from a naturally attenuated strain. The mutant clones showed reduced virulence in mice. Moreover, there were fewer specific CD8^+^ T cells targeting* T. cruzi*. Mice challenged with virulent parasites one year after the original infection with the mutant strain showed significant control over the secondary infection [15]. This study indicates that it is possible to generate genetically attenuated parasites that confer protection against new infections by* T. cruzi*.

Four doses of live parasites (Sylvio X10/4) administered at 3-week intervals were evaluated. One to two months after the last dose, the number of CD4^+^ T cells producing IFN-*γ* and memory cells and the proliferative response of T cells increased in the spleen. However, the challenge induced an increase in the serum IgG1 levels and mixed Th1/Th2 cytokine production. Moreover, there were no significant changes in heart injuries or subpatent parasitemias [16]. In conclusion, this study may help identify the elements necessary for a successful therapeutic vaccine that reduces human cardiomyopathy in chronically infected patients.

## 4. Cell Fraction Immunization

To identify the more immunogenic portion of the parasite that induces a protective immune response, a variety of cell fractionation studies have been performed.

Ruiz and colleagues obtained subcellular fractions of the Tulahuén strain of* T. cruzi* epimastigotes that were used to immunize mice. Subsequently, the mice were challenged with 25 blood trypomastigotes. The animals immunized with the pellet and supernatant showed positive xenodiagnosis, myocarditis, and myositis similar to the control animals.

Immunization with the flagellar portion resulted in partial protection against myocarditis development, fewer animals with positive xenodiagnosis, and no electrocardiographic changes. Fifty percent of animals immunized with the pellet or the supernatant, which were not challenged, had electrocardiographic changes and myocarditis. Immunization with the flagellar fraction in the absence of infection induced lesions similar to those in the control animals [17]. These data are troubling because they demonstrate that the fractions alone can produce damage, possibly by inducing an autoimmune response.

Wrightsman and colleagues used the paraflagellar rod protein (PAR) purified from* T. cruzi* epimastigotes to protect mice against a lethal inoculum of 10^3^ blood trypomastigotes. Subcutaneous injection of the PAR proteins reduced parasitemia and showed 100% survival after challenge. By contrast, the intraperitoneal route induced parasitemia levels equivalent to the control animals and the mice did not survive the infection [18]. One year later, the PAR protein was administered subcutaneously in combination with Freund's adjuvant or aluminum and the survival was 100% and 83%, respectively. The levels of IFN-*γ* and IL-2 were higher in the protective groups, indicating that the protective immunity in mice immunized with PAR was associated with a Th1-type response [19]. The induction of an immune response associated with the mucous, serous tissue, or spleen is not ideal.

## 5. Purified Protein Immunization

Immunization studies with purified proteins from the parasite to identify those that induce an immune response and provide protection to the challenge have been performed.

Snary compared the ability of two* T. cruzi* surface glycoproteins to confer protection against experimental infection. The 90 kDa glycoprotein found in all stages of* T. cruzi* protected against challenge with blood and metacyclic trypomastigotes. The 72 kDa glycoprotein found only in stages derived from insects protected against challenge with metacyclic trypomastigotes only [20]. These data indicate that selection of the immunogenic protein is important and must be present in the parasite stages that circulate in the mammal.

A protective study using 45 and 68 kDa antigens purified from the cell membrane of* T. cruzi* epimastigotes was performed by Araujo and Morein in 1991. The antigens were purified by affinity chromatography and incorporated into a system prepared with Quil A, a saponin derivative, for the immunization. A strong humoral and cellular response protected 100% of the immunized animals challenged with blood trypomastigotes [21].

Gomes and colleagues immunized C57B1/10 mice with an antigenic preparation called TcY 72, obtained when a lysate of* T. cruzi* Y strain epimastigotes was separated by electrophoresis and a 72 kDa band was isolated by electroelution. They observed the induction of high levels of IgG antibodies, a delayed hypersensitivity reaction after injection of epimastigotes in the legs of the mice, a significant reduction of parasitemia, and a decreased CD4/CD8 ratio in immunized mice [22], which suggest a possible role for CD8^+^ subpopulations.

Taibi and his group investigated the immunoprotective properties of trypomastigotes excretory-secretory antigens. The ESA immunization of BALB/c mice resulted in reduced parasitemia during acute infection and significant protection with 60% survival, whereas no mice in the control group survived after 39 days postinfection. The same experiments were performed in Fisher rats and showed greater protection against lethal infection with 100% survival [23].

The Gruppi group immunized mice with antigens released into the circulation, called exoantigens of pI 4.5, and observed partial protection. In subsequent experiments, they transferred the lymph node cells from immunized mice to normal recipients that were challenged, thereby reducing the parasitemia levels [24].

Garcia and colleagues administered* T. cruzi* soluble extract (TCSE) as an antigen in mice and analyzed the immune response when challenged with* T. cruzi*. The proliferative response in the animals upon stimulation with Concanavalin A was increased as was the IFN-*γ* level. The production of IFN-*γ* is important in controlling parasite replication because the protection by TCSE protecting was completely abrogated by* in vivo* treatment with a neutralizing anti-IFN-*γ* [25]. These data suggest that despite the antigenic complexity, it is possible to generate similar protection using a macromolecule or a set of macromolecules.

## 6. Recombinant Protein Immunization

The modern biotechnology allows obtaining many copies of a parasite gene (producing an immunogenic protein) inserted into the bacterial DNA in a short time and producing large quantities of recombinant proteins.

Pereira and his group immunized BALB/c mice subcutaneously three times with recombinant* T. cruzi* cytoplasmic repetitive antigen (CRA) and flagellar repetitive antigen (FRA). The recombinant CRA produced high IgG3 and IgG1 levels, whereas only IgG1 was induced by recombinant FRA [26]. Pereira and his group suggested that the IgG3 isotype plays significant role in protection. The complexity or structure of the antigen may generate a different humoral response. In Chagas disease, the humoral immune response is important because antibodies can protect animals, thereby reducing parasitemia and mortality.

Luhrs et al. produced recombinant paraflagellar rod proteins (PFR), and mice immunized with these proteins against* T. cruzi* showed protection associated with a Th1 cytokine profile. These authors analyzed the PFR-2 gene sequence in seven highly diverse strains of* T. cruzi* and found that it is highly conserved. Immunization with PFR-1, PFR-2, PFR-3, or an equimolar mixture induced a 70%, 73%, 51%, and 74% reduction in peak parasitemia, respectively. Of the animals immunized with PFR-3, 42% survived, whereas the remaining animals reached 100% survival [27]. It is important to identify new protective epitopes to optimize the design of vaccines.

Immunization of A/Sn mice with plasmid p154/13, which encodes a* T. cruzi trans*-sialidase, induced a Th1-type immune response. By contrast, immunization with the recombinant* trans*-sialidase generated a Th2-type immune response. Simultaneous administration of plasmid p154/13 and the recombinant protein in animals induced a Th2-type response, whereas when the first immunization was performed with two doses of plasmid followed by the recombinant protein, a Th1-type response was obtained as revealed by the reduction in the serum IgG1/IgG2a ratio and an increase of* in vitro* IFN-*γ* produced by CD4 T cells [28]. This study shows that immunization with DNA followed by immunization with a recombinant protein may increase the Th1-type response in DNA immunization protocols. The mechanism underlying these results is unclear; however, double stranded plasmid DNA may interact with toll-like receptors, stimulating the production of IL-12, which in turn stimulates the development of a Th1-type response.

Cazorla et al. inoculated mice with the C-terminal domain of cruzipain and high titers of IgG2a were obtained.* T. cruzi* invasiveness is blocked by antibodies from mice immunized with recombinant cruzipain and with the N-terminal domain. Mice immunized with the N-terminal domain and challenged with* T. cruzi* showed a lower concentration of enzymes associated with cardiomyopathy, such as creatine kinase and lactate dehydrogenase (LDH) [29]. This study shows that the N-terminal domain of cruzipain can produce a differential immune response, which may protect against a sublethal challenge with trypomastigotes.

Flores-García et al. evaluated the role of the* T. cruzi* recombinant protein rMBP::SSP4 with a GPI anchor as an immunomodulatory molecule. They showed the secretion* in vivo* of several cytokines such as IL-4, IL-10, IFN-*γ*, and TNF-*α*. The same cytokine profile was found* in vitro*, and besides IL-10 and IFN-*γ* were particularly secreted by CD4^+^ cells [30]. This cell population suppresses lymphocyte activity and plays an important role in regulating the immune response preventing a collateral damage generated by a strong immune response against the parasite, thus avoiding unwanted excessive tissue inflammation that would otherwise be exacerbated; this was proposed when mice were immunized with TcSP2-CHP and mice showed increased IL-2, IFN-*α*, and IL-10 cytokines after parasite change and protection in heart was observed [31]. In addition, it is involved in the prolonged persistence of the parasite and protection against severe inflammatory responses in the host.

A new antigen-adjuvant combination for protection against experimental Chagas disease was assessed. The antigen used in the formulation was a glycosylated mutant inactive* trans*-sialidase (mTS) and the adjuvant used was ISCOMATRIX (IMX). The mice immunized with mTS-IMX showed a TS-specific IgG response, increased IgG2a/IgG1 ratio, significant delayed-type hypersensitivity (DTH) reactivity, balanced production of IFN-*γ* and IL-10 by splenocytes, and strong IFN-*γ* secretion by CD8^+^ T lymphocytes. All mTS-IMX immunized infected mice showed ~50 times less parasitemia than nonimmunized infected mice; in the chronic phase, tissue presented ~4.5 times lower parasite load. These results indicate that mTS-IMX formulation induces both optimal humoral and cellular immune responses, conferring protection against* T. cruzi* [32].

## 7. DNA Vaccines against Chagas Disease

DNA vaccines provide an alternative for both prevention and treatment of a variety of infectious diseases [33–37], including Chagas disease.* TcSP* gene (encoding a member of the* trans*-sialidase superfamily),* TcSSP4* gene (encoding an amastigote-specific surface protein), or their recombinant proteins were used in an immunization protocol in murine model challenging with the H8 strain of* T. cruzi*. Immunization with the rTcSP recombinant protein or the* TcSP* gene gave a mixed Th1/Th2 T cell immune response. The mice vaccinated with* TcSSP4* showed significant amounts of IFN-*γ*, suggesting a Th1 response. Only the mice immunized with DNA showed a significant reduction in the parasitemia peak and lethal challenge survival [38, 39]. These studies demonstrate that DNA immunization induces a protective immune response in contrast to the use of homologous recombinant protein in the experimental infection with* T. cruzi.*

The prophylactic use of these two genes in Beagle dogs with Chagas disease showed that the dominant antibodies were subclass IgG2 immunoglobulins. It was also demonstrated that both genes' immunization induced cell mediated immunity characterized by lymphoproliferation as well as IFN-*γ* production [40]. Immunization decreased the quality and quantity of electrocardiographic abnormalities, thereby avoiding progression to more severe cardiac disturbances [41]. A partial protective effect for the prevention of macroscopic and microscopic damage in cardiac tissue during the chronic phase was also observed in these Beagle dogs [42]. These two genes of* T. cruzi* that generate a moderate level of protection in the chronic phase of the disease were proposed as vaccine candidates.

The immune response elicited by TcVac3 (antigenic candidates TcG2 and TcG4, membrane-associated GPI proteins) delivered by a DNA-prime/MVA-boost approach against challenge to the parasite in C57BL/6 mice has been characterized. The vaccine elicited a strong antigen- and parasite-specific, high avidity, lytic, Th1-type antibody response; it also elicited antigen- and parasite-specific CD4/CD8 T cell proliferation. The CD8^+^ T cells were predominantly IFN-*γ* ^+^ with cytolytic capacity. In chronic phase, immunized mice exhibited a massive decline in proinflammatory phenotype, a predominance of immunoregulatory IL-10^+^/CD4^+^ T and IL-10^+^/CD8^+^ T cells, and presented undetectable tissue parasitism, inflammatory infiltrate, and fibrosis [43]. In this study, the emergence of type 2 cytokine and T cell response suggested prevention of clinical disease.

The backbone of Yellow Fever (YF) 17D virus to express an immunogenic fragment derived from* T. cruzi* Amastigote Surface Protein-2 (ASP-2) was used. A/J mice were immunized subcutaneously with two doses of recombinant virus and four weeks after the last dose they were challenged with* T. cruzi*. Immunogenicity studies showed a reduction in mouse mortality, an increase of average survival time, and a reduction in peak parasitemia. The amount of IFN-*γ*-secreting splenocytes significantly increased for all mice immunized compared to the negative control group [44]. The results suggested expanding the applicability of YF 17D as a potential recombinant virus vector to express other antigens for Chagas disease vaccine development.

A DNA vaccine encoding TSA-1 and Tc24 antigens in a dog model of acute* T. cruzi* infection was evaluated. Mongrel dogs were immunized with two doses of 500 *μ*g of DNA vaccine and infected with SylvioX10/4 strain of* T. cruzi* two weeks after the second vaccine dose. Another group of dogs was infected first and treated with the vaccine. Both preventive and therapeutic vaccination significantly reduced parasitemia, cardiac inflammation, and cardiac parasite burden and tended to reduce the development of cardiac arrhythmias. The effect of vaccination on the immune response was weak because it did not observe the induction of a humoral response; however, an increased level of IFN-*γ* in immunized dogs was detected, suggesting that DNA vaccination tended to prime a cellular immune response [45]. These results demonstrated that the use of antigens expressed on DNA plasmids may be successful as preventive and therapeutic veterinary vaccine candidates in reducing both parasite transmission and the clinical progression of Chagas disease in vaccinated dogs and possibly other pets.

The TcG1, TcG2, and TcG4 antigens were delivered as a DNA prime/protein boost vaccine (TcVac2) in immunized C57BL/6 mice. Vaccinated mice showed lytic antibodies and type 1 CD8^+^ T cells that expanded upon challenge infection and provided >90% control of parasite burden and myocarditis in chagasic mice. Macrophages incubated with sera from vaccinated infected mice exhibited M2 surface markers (CD16, CD32, CD200, and CD206), moderate proliferation, a low oxidative/nitrosative burst as an indicator of low phagocyte cytotoxicity, and a regulatory/anti-inflammatory cytokine response (interleukin-4 [IL-4] plus IL-10 > tumor necrosis factor alpha [TNF-*α*]) [46]. The results suggested that the TcVac2 vaccine controls chronic myocarditis due to the antiproliferative and anti-inflammatory responses of macrophages. These findings open the possibility of using the* in vitro* phenotypic and functional profiling of macrophages to assess the efficacy of prophylactic and therapeutic vaccine candidates.

Vasconcelos et al. employed a heterologous prime-boost vaccination using plasmid DNA followed by replication-defective adenovirus vector (expressing the* ASP-2* gene) in the gzmBCreERT2/ROSA26EYFP transgenic mouse line. They observed that the frequency of the specific CD8^+^ T cells in the spleens of the vaccinated mice increased after challenge and the increase in the frequency of specific cells and the protective immunity they mediate were insensitive to treatment with the cytostatic toxic agent hydroxyurea. Specific CD8^+^ T cells were capable of producing simultaneously the antiparasitic mediators IFN-*γ* and TNF [47]. This group suggested that differentiation and recirculation, rather than proliferation, are key for the resultant protective immunity.

Cazorla et al. determined whether immunoprotection elicited by the cysteine protease cruzipain (Cz) could be improved by coadministration of* Salmonella* strains carrying plasmids encoding a thiol-transferase (Tc52) and a 24 kDa flagellar calcium-binding protein (FCaBP or Tc24) of* T. cruzi*. Immunized C3H/HeN mice with a multicomponent oral vaccine elicited strong humoral and cellular immune responses, conferring improved protection against* T. cruzi* infection with respect to monocomponent formulations [48]. This study demonstrates that the approach of protective effect searching the combination of several antigens involved in invasion or metabolic processes may highlight the benefits of a better immune response than vaccines with a single antigen.

The exploration of human vaccines against* T. cruzi* has been widely avoided due to the fear that this prophylactic measure could exacerbate the disease that many still consider to have an autoimmune etiology, although the evidence suggests that the best correlation between the induction and maintenance of inflammatory disease process is the persistence of the parasite in tissues of patients and not the immune response induced by the parasite [49].

The lack of financial support from governments and the pharmaceutical industry has been another factor that has greatly contributed to the disinterest in this field due to the fact that American trypanosomiasis is one of the diseases that affected the poorest people living in the Americas.

Chagas disease is included in the group of chronic parasitic, bacterial, and viral infections that actually promote poverty in people because they affect child development, pregnancy outcome, and worker productivity, so it is considered a Neglected Tropical Disease (NTD) [50].

Currently a therapeutic Chagas vaccine is under development by a consortium of Mexican (including the Carlos Slim Health Institute) and Texan scientific institutions; based on the evidence of therapeutic efficacy of Tc24 and TSA-1 vaccines in reducing* T. cruzi*-induced cardiac disease in mice and dogs, the authors are pursuing Tc24 and TSA-1 as lead candidate antigens. These researchers argued in 2012 that the two antigens comprising a prototype therapeutic vaccine for human Chagas disease could be produced under current Good Manufacturing Practices within the next five years [51].

## 8. Conclusions

The development of an effective human vaccine against Chagas disease has encountered many difficulties, and progress has been slow mainly because there is still controversy about its autoimmune etiology and disinterest of the authorities in endemic countries where this NTD exists. The genetic complexity of* T. cruzi* as well as the limited set of efficient engineering techniques for genome manipulating contributes significantly to the relative lack of progress in the understanding of this microorganism.

Despite several* T. cruzi* proteomic studies, the evaluation of bloodstream trypomastigotes profile remains unexplored. The approach of proteomic studies to develop vaccines that include antigens from both intracellular (amastigote) and extracellular (trypomastigote) forms is necessary.

The use of defined native antigens purified from* T. cruzi* is convenient and acceptable, but it is difficult to obtain these antigens in the volumes necessary to perform the studies. Monoclonal antibody production and advances in the methods for obtaining proteins have improved the identification, isolation, and purification of defined parasite proteins but increase the difficulty in achieving sufficient and sustainable vaccine production.

Obtaining antigens using molecular biology techniques and recombinant DNA have enabled the cloning, expressing, and production of* T. cruzi* antigens. These available molecular tools, combined with the knowledge of several parasite antigens, have allowed the development of new DNA vaccines. Furthermore, to enhance the immune response generated by DNA immunization with genes encoding molecules that stimulate the immune system, they have been coadministered with some* T. cruzi* genes.

Because there is no effective treatment for patients in acute stage, DNA could control* T. cruzi* infection and significantly reduce the progression of chronic Chagasic cardiomyopathy. The effectiveness of this type of vaccination is greater than conventional vaccine because DNA vaccines induce IFN-*γ* secretion and stimulate Th1 helper T cells, which are necessary to confer protection against infection with this parasite. However, it is important when selecting a vaccine containing genes or fragments thereof to demonstrate that the gene is present in the majority of* T. cruzi* strains, and if peptides are used, it must be demonstrated that the population immunized with this peptide expresses the correct MHC for presentation to CD4 or CD8 lymphocytes, as applicable. Besides, in chronically infected patients, it would be important to develop a therapeutic vaccine to induce the parasite complete elimination by the immune system.

The ideal prophylactic vaccination would be one that does not allow the parasite establishment or reduce the parasite burden, so it has a scientific basis for the use of immunization in humans and domestic reservoirs in endemic areas for prevention and control of Chagas disease.
